# The wild boar (*Sus scrofa*) Lymphocyte function-associated antigen-1 (CD11a/CD18) receptor: cDNA sequencing, structure analysis and comparison with homologues

**DOI:** 10.1186/1746-6148-3-27

**Published:** 2007-10-15

**Authors:** Philippe GAC Vanden Bergh, Laurent LM Zecchinon, Thomas Fett, Daniel JM Desmecht

**Affiliations:** 1 Pathology Department, Faculty of Veterinary Medicine, University of Liege, Colonster Boulevard 20 B43, B-4000 Liege, Belgium

## Abstract

**Background:**

The most predominant *beta*2-integrin lymphocyte function-associated antigen-1 (LFA-1, CD11a/CD18, *alpha*L*beta*2), expressed on all leukocytes, is essential for many adhesive functions of the immune system. Interestingly, RTX toxin-producing bacteria specifically target this leukocyte *beta*2-integrin which exacerbates lesions and disease development.

**Results:**

This study reports the sequencing of the wild boar *beta*2-integrin CD11a and CD18 cDNAs. Predicted CD11a and CD18 subunits share all the main structural characteristics of their mammalian homologues, with a larger interspecies conservation for the CD18 than the CD11a. Besides these strong overall similarities, wild boar and domestic pig LFA-1 differ by 2 (CD18) and 1 or 3 (CD11a) substitutions, of which one is located in the crucial I-domain (CD11a, E168D).

**Conclusion:**

As most wild boars are seropositive to the RTX toxin-producing bacterium *Actinobacillus pleuropneumoniae *and because they have sustained continuous natural selection, future studies addressing the functional impact of these polymorphisms could bring interesting new information on the physiopathology of *Actinobacillus pleuropneumoniae-*associated pneumonia in domestic pigs.

## Background

Cell adhesion receptors play crucial roles in multicellular organisms by mediating the direct cell/cell or cells/extracellular matrix proteins interactions. These molecular interactions condition the structural integrity of cells and tissues and contribute to the signalling transduction intervening in the cellular dynamic [1]. Cell adhesion receptors are subdivided in several membrane-associated protein families, including integrins, cadherins, immunoglobulin superfamily cell adhesion molecules, selectins, and syndecans. Integrins are a family of cell surface adhesion and signalling glycoproteins made up of non-covalently associated 120–180 kDa *α *and 90-110kDa *β *subunits [2]. There are 19 distinct *α *subunits and 8 *β *subunits that are combined to form 25 different heterodimeric receptors [1]. Each subunit possesses (i) a large extracellular N-terminal domain associating with that of the companion subunit to form the integrin headpiece which contains the ligand binding site, (ii) a single transmembrane stretch, and (iii) a short cytoplasmic C-terminal tail which mediates interactions with cytoskeleton and signalling proteins [1,3,4].

Within the integrin family, the leukocyte-specific *β*_2_-integrins (CD11/CD18) include four members sharing in common the *β*_2 _subunit (CD18) [5]: (i) the most predominant is the lymphocyte function-associated antigen-1 (LFA-1, CD11a/CD18, *α*_L_*β*_2_) on all leukocytes [6]; (ii) the CD11b/CD18 (Mac-1, CR3, *α*_M_*β*_2_) mainly on activated granulocytes and tissues macrophages [7]; (iii) the CD11c/CD18 (gp150/95, CR4, *α*_X_*β*_2_, Leu-M5) also on activated granulocytes and tissues macrophages [8], and (iv) the CD11d/CD18 (*α*_D_*β*_2_) abundant on the CD8+ lymphocyte and on macrophages [9-11]. These receptors are essential for an effective immune system as observed in repeated infections associated with the lymphocyte adhesion deficiency (LAD) type I syndrome, a disease due to mutations in the *β*_2 _(CD18) subunit gene leading to the lack of functional *β*_2 _integrins on the membrane surface of leukocytes [12-14].

The *β*_2_-integrin LFA-1 is essential for the following functions of the immune system [15-20] : (i) interaction between lymphocytes, (ii) interaction between T-cells and antigen presenting cells, (iii) adhesion of naïve lymphocytes to post-capillary high endothelial venules of secondary lymphoid tissues, (iv) adhesion of leukocytes to activated endothelium at sites of inflammation for extravasation, (v) control of cell differentiation and proliferation, and (vi) antibody-dependent killing by natural killer cells and granulocytes. Leukocyte LFA-1-mediated adhesion is engaged via the binding of the LFA-1 in an activated conformational state to membrane proteins, the so-called intercellular adhesion molecules (ICAM)-1 to -5 and the junctional adhesion molecule (JAM)-A [21].

Interestingly, several pathogens target the leukocytes *β*_2_-integrins which leads to lesions and disease development [22]. Several studies have highlighted the central role of LFA-1 in the pathogenesis of diseases caused by repeats-in-toxin (RTX)-producing bacteria. The virulence of *Aggregatibacter *(*Actinobacillus*) *actinomycetemcomitans *(localised aggressive periodontitis in humans), *Mannheimia haemolytica *(pneumonia in cattle), and pathogenic strains of *Escherichia coli *(extraintestinal infections) has been associated with a ligand/receptor interaction between their respective RTX toxin (LtxA, LktA, and HlyA) and the CD11a/CD18 receptor resulting in leukocytes alterations [23-26]. This interaction triggers synthesis and release of a wide array of cytokines and chemoattractants by the leukocytes that exacerbate inflammation and ultimately results in a much greater leukolysis worsening the lesions [25,27]. *Actinobacillus pleuropneumoniae*, a causative agent of pleuropneumonia in domestic pigs (*Sus scrofa domestica*), responsible for economic losses and antibiotic usage in the pork industry, also produces RTX toxins (ApxIA, -IIA, -IIIA, and -IVA) [28,29]. We therefore hypothesize that the pathogenesis of this disease similarly relies on an interaction with the *Sus scrofa domestica *LFA-1, whose CD11a (*α*_L_) and CD18 (*β*_2_) subunits have been well characterised [30,31]. On the basis of the report that approximately 50% of wild boars in their natural environment are serologically positive for *Actinobacillus pleuropneumoniae *[32] and because these wild pigs sustain losses due to natural selection pressure, we hypothesize that some LFA-1 molecular peculiarities conferring resistance to wild boars could have been selected. In this context, the purpose of this study was to report the sequence and analysis of the cDNAs encoding wild boar LFA-1 (WbCD11a/WbCD18) and to point out the wild boar LFA-1 specificities that might confer resistance to *Actinobacillus pleuropneumoniae*-associated pneumonia.

## Results and discussion

### Characterization of WbCD11a-encoding cDNA and deduced amino-acid sequence

**Figure 1:**
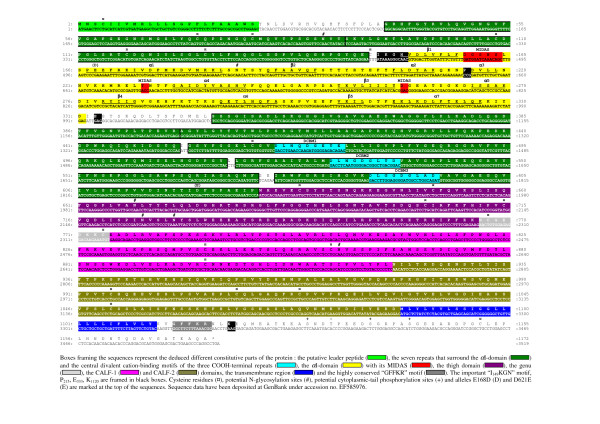
The nucleotide and deduced amino acid sequences of wild boar CD11a cDNA.

The WbCD11a cDNA sequence contains an ORF of 3519 bp [GenBank:EF585976] that codes for 1172 aa (Fig. 1). Starting from the N-terminal end, the 1172 aa mature WbCD11a contains a 23-residue putative leader peptide (M_1_-S_23_), an extracellular domain of 1064 residues (Y_24_-D_1086_), a single hydrophobic transmembrane region of 24 residues (M_1087_-Y_1110_) and a short cytoplasmic tail of 62 residues (K_1111_-A_1172_) (Fig. 1). Six N-linked putative glycosylation sites (Asn-Xaa-Ser/Thr) are found in the extracellular domain (Fig. 1). The WbCD11a possesses 22 cysteine residues, among which one is located in the cytoplasmic tail (Fig. 1). A subset of integrin *α *chains (*α*_1_, *α*_2_, *α*_10_, *α*_11_, *α*_D_, *α*_E_, *α*_L_, *α*_M _and *α*_X_), including CD11a, contains a I-domain (for Inserted domain, also called *α*_L_I-domain or *α*_L_A-domain) that is homologous to the family of von Willebrand Factor (vWF) A-type domains and to cartilage matrix protein [33,34]. The I-domain has been associated with ligand binding. Its three-dimensional structure consists of a five-stranded parallel *β*-sheet core surrounded on both faces by seven *α*-helices (Fig. 1). A short antiparallel strand occurs on one edge of this sheet [35]. The I-domain (I_149_-D_331_) contains a metal ion-dependent adhesion site (MIDAS) (residues D_160_-S_164_, T_229_, D_262_) [35,36] (Fig. 1). The I-domain crystallisation has demonstrated that a "closed" (low affinity) and an "open" (high affinity) forms exist and that the major conformational changes during transition from the closed to open states include a rearrangement of the cation-coordinating residues in the MIDAS site, accompanied by a small inward movement of the *α*1 helix and a large downward shift of the mobile C-terminal *α*7 helix [37]. The extracellular domain of WbCD11a contains seven internal repeats (FG-GAP) (G_40_-T_89_, S_90_-E_147_, S_348_-R_398_, A_399_-Q_453_, G_455_-D_511_, G_513_-F_573_, I_576_-P_628_) that surround the I-domain (Fig. 1) [38,39]. The degree of identity is highest among the three COOH-terminal repeats (18–31%) and their central region (D_466_-E_474_, D_528_-D_536 _and D_588_-D_596_) is similar to the EF hand divalent cation-binding motifs (DCBM) of troponin C, parvalbumin and galactose binding protein [38] (Fig. 1). All the cysteine residues and all but one N-glycosylation sites are found outside the I-region and divalent cation binding motifs (Fig. 1), consistent with the hypothesis that these regions may undergo conformational changes important in ligand binding [38,40]. Between the FG-GAP 7 and the transmembrane domain stands the thigh domain (M_629_-K_766_), the genu (N_767_-C_774_), and the CALF domains (E_775_-L_920_, N_921_-D_1086_) [41]. The cytoplasmic portion of WbCD11a contains four potential phosphorylation sites and also a conserved "G_1113_FFKR" basic sequence near the transmembrane region (Fig. 1). The integrins become constitutively active when this sequence is deleted. The "G_1113_FFKR" motif thus normally fixes the integrins in an inactive state [11,42].

**Figure 2:**
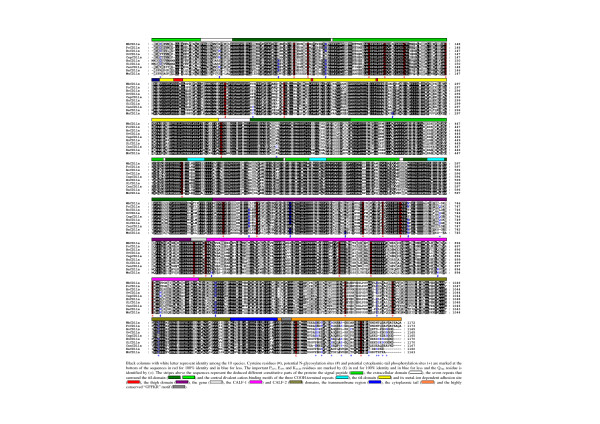
Comparison of the wild boar (Wb-), porcine (Po-), bovine (Bo-), ovine (Ov-), caprine (Cap-), human (Hu-), simian (Si-), canine (Can-), rat (Ra-) and murine (Mu-) α_L _subunits amino acids sequences.

Beside the complex mechanisms of affinity/avidity regulation of the integrins, the existence of several isoforms issued from alternative splicing complicates the biological understanding of these glycoproteins [43]. Previously, we have characterised two different forms of PoCD11a due to the presence of a supplementary "cag" codon that codes for a glutamine (Q) in position 744 [GenBank:DQ013284, GenBank:DQ013285] [31]. The addition of a Gln at the same position was also observed in the human (Q_746_) [GenBank:NM_002209, GenBank:AY892236], the simian (Q_746_) [44], ovine (Q_743_) [45] and caprine (Q_743_) [GenBank:AY773018, GenBank:AY773019] CD11a cDNAs (Fig. 2). This addition located in the thigh domain of the extracellular part of CD11a, just above the genu, increases the length of an *α*-helix in the PoCD11a according to the GORIV bioinformatic program. Until now, it was not clear whether this addition represented two alleles or was generated by an alternative splicing. We have recently cloned and sequenced a third PoCD11a form characterised by an insertion of 27 amino acids at the same position (P_744_EPLQLSSTSSAASATLSRLPLLCAQ**Q**_770_) [GenBank:DQ474234] which is predicted to lengthen the *α*-helix further. The nucleotidic sequence of this insertion corresponds to that of the 3'end of the adjacent bovine and human intron 18 (79% and 70% of identity respectively), suggesting that the insertion of the glutamine or of the 27 amino acids-long stretch in position 744 of the thigh domain comes from an alternative splicing rather than from different alleles. Although these two insertions were not observed in the WbCD11a yet and because of the between-species conservation of this potential alternative splicing site, we hypothesize that it can have a biological importance for the mature CD11a, for example, in regulating the ligand binding and signaling activity.

### WbCD11a comparison among species

Overall, the general organization of wild boar (*Sus scrofa*), porcine (*Sus scrofa domestica*) [31], bovine (*Bos taurus*) [46], ovine (*Ovis aries*) [45], caprine (*Capra hircus*) [47], human [38], simian (*Pan troglodytes*) [44], canine (*Canis familiaris*) [GenBank:XM_547024], rat (*Rattus norvegicus*) [GenBank:NP_001029170], and murine (*Mus musculus*) [48] CD11a proteins is quite similar (Fig. 2). Comparison between mature WbCD11a sequence and its porcine, bovine, ovine, caprine, human, simian, canine, rat and murine counterparts shows respectively overall 99%, 77%, 77%, 77%, 76%, 76%, 76%, 70% and 69% identity, and 99%, 87%, 86%, 86%, 86%, 86%, 85%, 81%, and 80% similarity (BLOSUM62table) (Table 1). The highest identity is found for the "G_1113_FFKR" motif, the genu, the MIDAS motif and the transmembrane region and the lowest for the cytoplasmic tail and the putative signal peptide (Table 1). Although DCBM3 presents a weak identity, its similarity score is high. The "G_1113_FFKR" sequence is highly conserved which is consistent with the stabilizing role of this motif for the *alpha*/*beta *complex, possibly because of its direct involvement in heterodimer formation [42]. The genu seems to play a key role in the activation of the LFA-1 through the deployment of the receptor [41] and its great conservation is therefore not surprising. The high conservation of the MIDAS and the putative cation binding motifs is consistent with an involvement of these regions in the functional activity of the LFA-1 *α *subunit, as suggested by the requirement of Mg^2+ ^and Ca^2+ ^for CD11a/CD18-dependent cellular interactions [40] or binding to purified ICAM-1 [49,50]. The transmembrane region also shows a high degree of conservation, probably due to shared physicochemical and functional constraints. Indeed, residues lying in the membrane first have to possess a hydrophobic character to warrant liposolubility, which is confirmed by the presence of many leucine residues (Fig. 2). Secondly, bidirectional integrin signalling (inside-out and outside-in) is accomplished by transmission of information across the plasma membrane [51]. By contrast, the low conservation of the COOH-terminal part of the cytoplasmic tail suggests that it is not required to guarantee adequate functioning of LFA-1. This is in agreement with the observation that truncation of the LFA-1 *α *subunit cytoplasmic domain has no effect on binding to ICAM-1, whereas binding is markedly diminished by *β *subunit cytoplasmic domain truncation [52]. The "I_149_KGN" motif known to participate in the binding to ICAM-3 [53] shows a high degree of conservation (Fig. 2). The amino acid P_215_, participating to the binding to ICAM-1 [36] is highly conserved too (Fig. 2). Residue E_333_, located in the linker following the I domain which is critical for communication with the *β*_2 _I-like domain, rolling, integrin extension and activation by Mn^2+ ^[16] is logically strictly conserved too. The K_1120 _residue, critical for Rap1-dependent LFA-1 activation and affinity up-regulation [5] is also strongly conserved (Fig. 2). Every cysteine residue in the mature WbCD11a is present at the same location in bovine, ovine, human, simian, and murine CD11a, which is consistent with a role in maintaining the global structure of the protein. Finally, of six potential Asn-glycosylation sites in WbCD11a, the ones present at amino acids 186 and 724 are strictly conserved (Fig. 2). In addition, although WbCD11a sequences were obtained from only four wild boars, one of them was heterozygous. Both alleles differed from those found in pigs by a G736A substitution. One allele displayed 2 additional substitutions compared to pigs: E168D (in the I-domain) and D621E (in the FG-GAP7, Fig. 1). According to the BLOSUM 62 table, these substitutions are theoretically predicted to have a weak impact on the general structure of CD11a. However, these two wild boar-specific CD11a isoforms might display an altered/improved function compared to those described among domestic pigs [45].

**Table 1:** Between-species percent identities and similarities of CD11a constitutive blocks

**Block**	**Wb vs. Po** **(%)**	**Wb vs. Bo** **(%)**	**Wb vs. Ov** **(%)**	**Wb vs. Cap** **(%)**	**Wb vs. Hu** **(%)**	**Wb vs. Si** **(%)**	**Wb vs. Can** **(%)**	**Wb vs. Ra** **(%)**	**Wb vs. Mu** **(%)**
**Overall**	99%^(1)^ 99%^(2)^	77% 87%	77% 86%	77% 86%	76% 86%	76% 86%	76% 85%	70% 81%	69% 80%

Putative signal peptide	100% 100%	86% 95%	78% 86%	78% 86%	82% 91%	56% 68%	69% 73%	50% 54%	54% 58%

Extracellular domain	99% 99%	79% 87%	78% 87%	78% 87%	78% 87%	78% 87%	77% 87%	72% 82%	71% 82%
FG-GAP 1	100% 100%	80% 86%	74% 82%	74% 82%	80% 84%	80% 84%	76% 86%	66% 80%	56% 76%
FG-GAP 2	100% 100%	86% 93%	86% 91%	86% 91%	84% 89%	84% 89%	84% 87%	77% 81%	81% 82%
I-domain	99% 100%	82% 89%	82% 90%	82% 90%	80% 90%	80% 90%	82% 87%	75% 85%	72% 85%
MIDAS	100% 100%	85% 85%	85% 85%	85% 85%	85% 85%	85% 85%	85% 85%	85% 85%	85% 85%
FG-GAP 3	100% 100%	82% 90%	80% 86%	82% 88%	86% 94%	86% 94%	78% 86%	76% 84%	80% 88%
FG-GAP 4	100% 100%	85% 90%	83% 87%	85% 89%	74% 83%	76% 83%	80% 85%	72% 78%	70% 78%
FG-GAP 5	100% 100%	78% 93%	76% 91%	76% 91%	83% 95%	83% 95%	83% 95%	80% 91%	80% 90%
DCBM 1	100% 100%	66% 100%	66% 100%	66% 100%	77% 100%	77% 100%	77% 100%	77% 100%	77% 88%
FG-GAP 6	100% 100%	82% 86%	81% 86%	82% 86%	79% 86%	77% 86%	81% 87%	81% 89%	87% 91%
DCBM 2	100% 100%	77% 88%	77% 88%	77% 88%	77% 88%	77% 88%	77% 88%	88% 100%	77% 88%
FG-GAP 7	98% 100%	90% 96%	90% 96%	90% 96%	83% 90%	83% 90%	90% 92%	84% 88%	81% 88%
DCBM 3	100% 100%	100% 100%	100% 100%	100% 100%	88% 88%	100% 100%	88% 88%	88% 88%	88% 88%
Thigh domain	98% 98%	80% 87%	79% 87%	79% 87%	79% 84%	79% 84%	74% 84%	71% 84%	67% 81%
Genu	100% 100%	87% 87%	87% 87%	87% 87%	100% 100%	100% 100%	100% 100%	100% 100%	100% 100%
CALF 1	100% 100%	67% 83%	67% 81%	67% 81%	67% 80%	67% 81%	67% 79%	61% 73%	62% 73%
CALF 2	100% 100%	71% 81%	72% 83%	71% 81%	77% 89%	77% 89%	71% 88%	68% 83%	69% 83%

Transmembrane region	100% 100%	83% 91%	87% 91%	87% 91%	91% 95%	91% 95%	79% 91%	83% 87%	75% 91%

Cytoplasmic tail	100% 100%	50% 69%	50% 70%	50% 69%	51% 64%	51% 64%	54% 67%	42% 52%	44% 52%
"GFFKR" motif	100% 100%	100% 100%	100% 100%	100% 100%	100% 100%	100% 100%	100% 100%	100% 100%	100% 100%

Wb, Po, Bo, Ov, Cap, Hu, Si, Can, Ra, and Mu: wild boar, porcine, bovine, ovine, caprine, human, simian, canine, rat, and murine CD11a, respectively ; FG-GAP : β-propeller repeat; MIDAS: metal-ion dependent adhesion site ; DCBM : divalent-cation binding motif ; vs : versus. (1) identity; (2) similarity (BLOSUM62 table).

### Characterization of WbCD18-encoding cDNA and deduced amino-acid sequence

**Figure 3:**
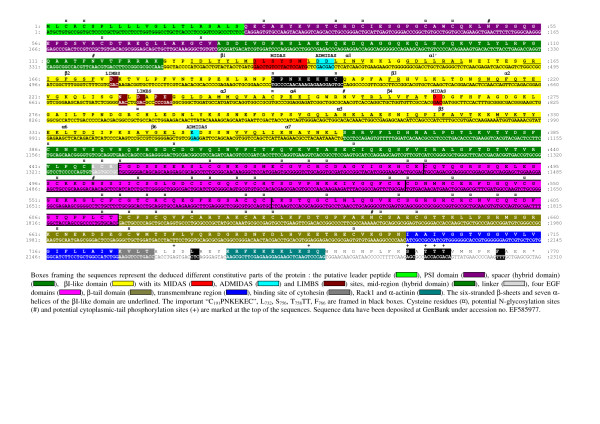
The nucleotide and deduced amino acid sequences of wild boar CD18 cDNA.

The cDNA sequence of WbCD18 contains an ORF of 2310 bp [GenBank:EF585977] that codes for 769 aa (Fig. 3). The mature WbCD18 contains a 22 aa putative leader peptide (M_1_-S_22_), an extracellular domain of 679 residues (Q_23_-N_700_), a single hydrophobic transmembrane region of 23 residues (I_701_-W_723_) and a short cytoplasmic tail of 46 residues (K_724_-R_769_) (Fig. 3). Starting from the N-terminal end, the extracellular region successively contains a cysteine-rich repeats-containing analogue of the so-called [54] plexin-semaphorin integrin domain (Q_23_-V_74_) [55], a spacer (A_75_-K_123_) [55,56], an inserted I-like domain of 240 amino acids (G_124_-L_363_), a mid-region (S_364_-C_445_) that fold-up with the spacer to form the hybrid domain [55,56], a cysteine-rich region containing four EGF-like regions (C_449_-E_482_, C_483_-E_535_, C_536_-Q_574_, and C_575_-T_613_) [39,55,57], and a C-terminal *β*-tail domain [5] (D_614_-N_700_) (Fig. 3). A putative metal ion-dependent adhesion site (MIDAS)-like DXSXS motif (D_134_-M_139_), an adjacent site to MIDAS (ADMIDAS) (D_141_, D_142_, E_347_), and a ligand-induced metal-binding site (LIMBS) (D_173_, N_229_, D_231_, P_233_, E_234_) motif are predicted within the I-like domain [58]. The cytoplasmic tail contains the binding sites for cytohesin, Rack1 (K_724_VLT), and *α*-actinin (K_736_RFEKEKLKSQ) [59]. Overall, the protein contains 58 cysteine residues and 5 N-linked putative glycosylation sites (Asn-X-Thr/Ser), all located within the extracellular region (Fig. 3).

### WbCD18 comparison among species

**Figure 4:**
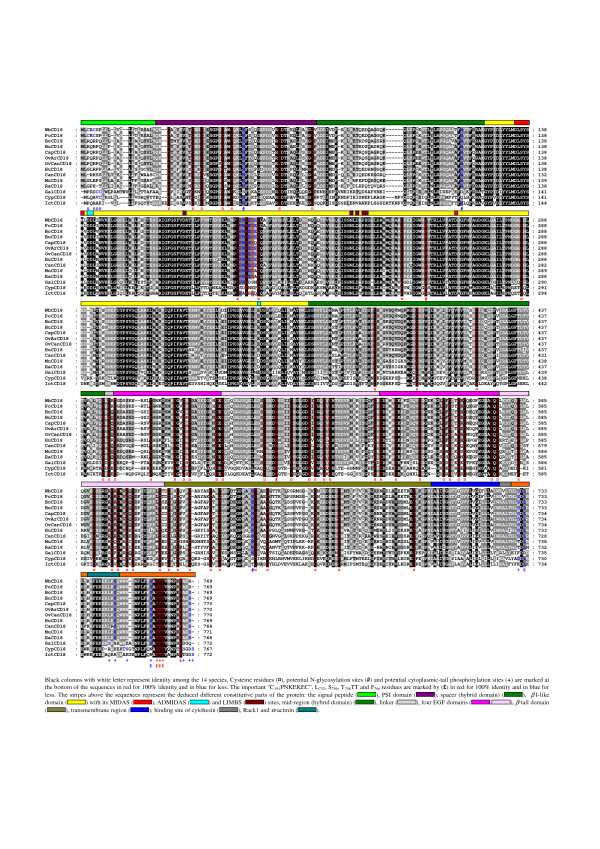
Comparison of the wild boar (Wb-), porcine (Po-), bovine (Bo-), water buffalo (Bu-), caprine (Cap-), ovine (*Ovis aries *and *canadensis*) (OvAr- and OvCan-), human (Hu-), canine (Can-), murine (Mu-), rat (Ra-), chicken (Gal-), carp (Cyp-) and channel catfish (Ict-) β_2 _subunits amino acids sequences.

Overall, the general organization of wild boar (*Sus scrofa*), porcine (*Sus scrofa domestica*) [30], bovine (*Bos taurus*) [60], water buffalo (*Bubalus bubalis*) (GenPept AAW29104), caprine (*Capra hircus*) [61], ovine (*Ovis aries *and *Ovis canadensis*) [62,63], human [64], canine (*Canis familiaris*) [65], murine (*Mus musculus*) [66], rat (*Rattus norvegicus*) [GenBank:NM_001037780], chicken (*Gallus gallus*) [67], carp and channel catfish (*Cyprinus carpio *and *Ictalurus punctatus*) [GenBank:AB031070] [68] CD18 proteins is quite similar (Fig. 4). Sequence comparisons between WbCD18 and its porcine, bovine, water buffalo, caprine, ovine, human, canine, murine, rat, chicken, carp and channel catfish counterparts shows respectively, 99%, 88%, 88%, 88%, 88%, 87%, 83%, 81%, 81%, 80%, 62%, 49% and 48% identity, and 99%, 93%, 93%, 93%, 93%, 93%, 90%, 89%, 88%, 88%, 76%, 64% and 63% similarity (BLOSUM62 table) (Table 2). The MIDAS-like, ADMIDAS, LIMBS motifs, the I-like domain, the EGF-2 domain and the cytoplasmic tail have the highest identity while the putative peptide signal, the *β*-tail domain, and the EGF-1 show the lowest identity (Table 2). The very high interspecies conservation of the putative MIDAS-like, ADMIDAS, LIMBS, I-like domains and the cytoplasmic tail is consistent with an involvement of these regions in the functional activities of *β*_2_-integrins. Overall, the high evolutionary conservation of the I-like domain confirms its importance in *β*_2_-integrins functions, which is compatible with the observation that monoclonal antibodies binding epitopes mapped within this region inhibit binding of LFA-1 to ICAMs 1–3 [56]. The maximum conservation being observed for the CD18 MIDAS-like motif, it is tempting to speculate that it plays a fundamental role in *β*_2_-integrin function. In this way, it was demonstrated that the C_169_PNKEKEC sequence conserved among mammalian species (Fig. 4) constitutively activates LFA-1 binding to ICAM-1 [69]. LIMBS and ADMIDAS sites modulate binding of ligand to the MIDAS-like in the integrins that lack the *α*I domain [70-72] but the ADMIDAS seems also to regulate *α*_L _I domain affinity and to participate in the outside-in signalling [58]. The high degree of conservation in the cytoplasmic tail, with many Ser, Thr, and Tyr residues, is compatible with the important role that phosphorylation of these residues plays in regulating adhesive activity [73] and with the observation that cytoplasmic domain truncation of CD18 markedly diminishes binding of LFA-1 to ICAM-1 [52]. Importantly, it was shown that the phosphorylation of the highly conserved residues T_758_, T_759 _and T_760 _plays a crucial role in the activation of the receptor and the binding to ICAM-1 [59,74]. In addition, the key residue F766 for binding to ICAM-1 is strictly conserved [59]. Although EGF-1 possesses a weaker identity, its similarity is very high (Table 2). The weaker conservation of the *β*-tail domain could translate a less degree of importance of this domain for the CD18 function. Every cysteine residue in the wild boar extracellular portion of mature CD18 is present at the same location in CD18 from other species, which is consistent with a role in maintaining the global structure of the protein. Similarly, all five potential Asn-glycosylation sites observed in wild boar are present at the same location in other mammalian species. Wild boar-specific CD18 isoform is characterized by two amino acid substitutions compared to domestic pigs: G560S and A721V, which should not result in structural differences, according to BLOSUM 62 table, but might impact CD18 function.

**Table 2:** Between-species percent identities and similarities of CD18 constitutive blocks

**Block**	**Wb vs. Po** **(%)**	**Wb vs. Bo** **(%)**	**Wb vs. Bu** **(%)**	**Wb vs. Cap** **(%)**	**Wb vs. OvAr** **(%)**	**Wb vs. OvCan** **(%)**	**Wb vs. Hu** **(%)**	**Wb vs. Can** **(%)**	**Wb vs. Mu** **(%)**	**Wb vs. Ra** **(%)**	**Wb vs. Gal** **(%)**	**Wb vs. Cyp** **(%)**	**Wb vs. Ict** **(%)**
**Overall**	99%^(1)^ 99%^(2)^	88% 93%	88% 93%	88% 93%	88% 93%	87% 93%	83% 90%	81% 89%	81% 88%	80% 88%	62% 76%	49% 64%	48% 63%

Putative signal peptide	100% 100%	59% 68%	54% 68%	59% 68%	59% 72%	59% 72%	56% 60%	56% 60%	52% 60%	60% 60%	28% 44%	28% 32%	22% 50%

Extracellular domain	99% 99%	88% 94%	89% 94%	89% 94%	88% 94%	88% 93%	84% 91%	82% 91%	81% 89%	80% 88%	63% 77%	50% 64%	48% 63%
PSI	100% 100%	84% 92%	86% 94%	88% 92%	88% 92%	86% 90%	75% 88%	88% 92%	80% 90%	78% 88%	65% 78%	55% 69%	61% 75%
Spacer (Hybrid domain)	100% 100%	77% 91%	79% 91%	77% 93%	77% 93%	77% 93%	89% 93%	83% 93%	79% 87%	67% 79%	38% 56%	25% 42%	30% 50%
I-like domain	99% 100%	96% 98%	96% 98%	95% 98%	95% 98%	95% 98%	96% 98%	95% 99%	95% 97%	95% 97%	79% 89%	65% 75%	63% 75%
MIDAS	100% 100%	100% 100%	100% 100%	100% 100%	100% 100%	100% 100%	100% 100%	100% 100%	100% 100%	100% 100%	100% 100%	100% 100%	100% 100%
ADMIDAS	100% 100%	100% 100%	100% 100%	100% 100%	100% 100%	100% 100%	100% 100%	66% 100%	66% 100%	66% 100%	100% 100%	100% 100%	66% 66%
LIMBS	100% 100%	100% 100%	100% 100%	100% 100%	100% 100%	100% 100%	100% 100%	100% 100%	100% 100%	100% 100%	100% 100%	100% 100%	100% 100%
Mid-region (Hybrid domain)	100% 100%	91% 96%	91% 96%	93% 96%	93% 96%	92% 95%	92% 97%	89% 95%	84% 90%	82% 90%	58% 71%	41% 53%	32% 54%
EGF-1	97% 100%	73% 91%	70% 91%	76% 94%	73% 94%	70% 91%	67% 82%	61% 79%	70% 88%	67% 85%	35% 52%	47% 64%	30% 52%
EGF-2	100% 100%	100% 100%	100% 100%	98% 100%	98% 100%	98% 100%	88% 96%	81% 94%	88% 98%	88% 98%	79% 94%	41% 58%	41% 50%
EGF-3	97% 97%	87% 94%	87% 94%	87% 92%	82% 92%	79% 92%	76% 87%	69% 87%	66% 79%	79% 84%	64% 76%	48% 66%	51% 74%
EGF-4	100% 100%	92% 94%	92% 94%	92% 92%	92% 92%	92% 92%	69% 82%	76% 92%	66% 76%	69% 79%	61% 66%	43% 58%	38% 48%
β-tail domain	100% 100%	74% 81%	74% 81%	73% 80%	73% 80%	73% 80%	57% 71%	54% 65%	56% 69%	56% 70%	42% 64%	39% 52%	39% 56%

Transmembrane region	95% 95%	91% 95%	91% 95%	91% 95%	95% 100%	95% 100%	78% 91%	82% 91%	73% 91%	69% 91%	56% 73%	52% 78%	52% 78%

Cytoplasmic tail	100% 100%	93% 93%	93% 93%	93% 93%	93% 93%	93% 93%	91% 93%	89% 95%	91% 95%	89% 93%	65% 82%	50% 68%	50% 68%

Cytohesin, Rack1 binding site	100% 100%	75% 75%	75% 75%	75% 75%	75% 75%	75% 75%	50% 50%	75% 75%	75% 75%	75% 75%	50% 100%	25% 50%	50% 50%
α-actinin binding site	100% 100%	90% 90%	90% 90%	90% 90%	90% 90%	90% 90%	90% 100%	90% 100%	90% 100%	90% 100%	63% 90%	45% 72%	54% 72%

Wb, Po, Bo, Bu, Cap, OvAr, OvCa, Hu, Can, Mu, Ra, Gal, Cyp, and Ict: wild boar, porcine, bovine, water buffalo, caprine, ovine (*ovis aries*), ovine (*ovis canadensis*), human, canine, murine, rat, chicken, carp, and channel catfish CD18, respectively ; PSI : plexin-semaphorin integrin ; MIDAS: metal-ion dependent adhesion site ; ADMIDAS : adjacent to MIDAS ; LIMBS : ligand-induced metal-binding site ; EGF : epidermal growth factor site; vs : versus. (1) identity; (2) similarity (BLOSUM62 table).

## Conclusion

This study reports the sequencing of the wild boar *β*_2_-integrin CD11a/CD18 subunits cDNAs. Predicted CD11a and CD18 subunits share all the main structural characteristics of their mammalian homologues, with a larger interspecies conservation for the CD18 than the CD11a. Besides these strong overall similarities, wild boar and domestic pig LFA-1 differ by 2 (CD18) and 1 or 3 (CD11a) substitutions, of which one is located in the crucial I-domain (CD11a, E168D). As most wild boars are seropositive to *Actinobacillus pleuropneumoniae *and because they have sustained continuous natural selection, future studies of the functional impact of these polymorphisms could bring interesting new information on the physiopathology of pneumonia in domestic pigs.

## Methods

### RNA isolation

Total RNA from spleen of freshly slaughtered wild boars (*Sus scrofa*) was extracted with TRIzol (Invitrogen, USA) as described by the manufacturer.

### Amplification and sequencing of the full length cDNA

Total RNA from spleen was reverse transcribed using Improm II (Promega). The full-length cDNA was then generated by long distance PCR using *Taq *and *Pfu *DNA polymerase from the High Fidelity PCR Enzyme Mix (Fermentas) with primers designed from the 5'- and 3'UTR of PoCD11a [31] and PoCD18 [30]. The couple of primers used for amplification of WbCD11a and WbCD18 are the next: 5'GGTATGGTCCCTCCAGAAGC-3' (CD11a forward), 5'-GCAGGCTGAGTCCAGTCCTG-3' (CD11a reverse), 5'-GAGGTCTCCAGGACATCAAG-3' (CD18 forward) and 5'-TAGGGGTGCTTGGTGAAGAC-3' (CD18 reverse). The procedures recommended by the manufacturer were applied, with the following cycling parameters : 5 min at 94°C, then 35 cycles including (i) 30 s at 94°C, (ii) 45 s at 60°C and (iii) 3 min 45 s at 72°C, and a final extension at 72°C for 10 min. Resulting PCR products were purified using the NucleoSpin^®^ExtractII kit (Macherey-Nagel), and sequenced on a ABI-3100 Genetic Analyzer using the Big Dye terminator chemistry (Applied Biosystems). The CD11a/CD18 cDNA sequences were deduced from sequences obtained from four independent wild boars. Sequences data have been deposited at GenBank under accession nos. EF585976 and EF585977.

### Bioinformatics

Primers design was performed with Netprimer [75] and Primer 3 [76]. Nucleotidic sequence and identity analyses were carried out using respectively Chromas v.2.21 [77] and BLAST programs [78]. Alignment of amino acids sequences was drawn by GeneDoc v.2.6.002 [79] following the BLOSUM 62 matrix. SignalP v.2.0.b2 [80] and NetNGlyc v.1.0 [81] provided peptide signal and N-glycosylation sites prediction, respectively. The secondary structures were resolved by the GOR secondary structure prediction method version IV [82].

## List of abbreviations

aa, amino acid ; ADMIDAS, adjacent to MIDAS ; Bo, bovine; Can, canine ; Cap, caprine ; CD, cluster of differentiation ; CR, complement receptor ; Cyp, *Cyprinus *; DCBM, divalent-cation binding motif ; EGF, epidermal growth factor ; Gal, *Gallus *; Hu, human; ICAM, intercellular adhesion molecule ; Ict, *Ictalurus*; LFA, lymphocyte function-associated antigen; LIMBS, ligand-induced metal-binding site; MIDAS, metal-ion dependent adhesion site; Mu, murine; Ov, ovine ; OvAr, *Ovis aries *; OvCan, *Ovis Canadensis *; Po, porcine ; Ra : rat ; PSI, plexin-semaphorin integrin ; Si : simian, Wb : wild boar.

## Authors' contributions

PVB carried out amplification, sequencing, sequences alignment and drafting of the manuscript. TF and LZ participated in the design of the study and helped to structure analysis. DD participated in the design of the study and coordination and helped to draft the manuscript. All authors read and approved the final manuscript.
